# Editorial: Advances in Human Immune System Mouse Models for Studying Human Hematopoiesis and Cancer Immunotherapy

**DOI:** 10.3389/fimmu.2021.829644

**Published:** 2021-12-23

**Authors:** Yasuyuki Saito, Tim Willinger, Alexandre P. A. Theocharides

**Affiliations:** ^1^ Division of Molecular and Cellular Signaling, Department of Biochemistry and Molecular Biology, Kobe University Graduate School of Medicine, Kobe, Japan; ^2^ Center for Infectious Medicine, Department of Medicine Huddinge, Karolinska University Hospital, Karolinska Institutet, Stockholm, Sweden; ^3^ Department of Medical Oncology and Hematology, University of Zurich and University Hospital Zurich, Zurich, Switzerland

**Keywords:** humanized mouse, hematopoiesis, cancer immunotherapy, human immunology, translational research

## Introduction

Gaining knowledge of human physiology and pathophysiology is often hampered by restricted access to human tissues or limited to performing *in vitro* assays. Furthermore, the development of novel therapeutics for cancer immunotherapy, autoimmune- and inflammatory diseases is tightly restricted by the use of human samples before moving to clinical trials, which is generally slow and costly. Human Immune System (HIS) mice - immunodeficient mice reconstituted with a human immune system – offer the unique opportunity to comprehensively study human hematopoiesis, infectious diseases, autoimmunity, and anti-tumor immunity *in vivo*. Recent progress in HIS mouse models, in which recipient immunodeficient mice carry several gene mutations or express human cytokines and self-recognition molecules, have improved human hematopoietic stem and progenitor cell (HSPC) engraftment as well as functional immune cell development in primary and secondary lymphoid tissues.

The articles in this Research Topic describe the latest developments in the field and indicate future directions to further improve HIS models and to apply them for more precisely characterizing human hematopoiesis as well as the human immune response against cancer.

## Recent Progress in HIS Mouse Models

Human cell lines and organoids have proven to be particularly useful for *in vitro* high-throughput screens or gain/loss of function library studies in order to identify novel therapeutic targets for human diseases. The advantage of *in vivo* models is the more faithful recapitulation of human disease. Xenograft models support the development of human disease, the assessment of patient heterogeneity, and the investigation of the human immune system *in vivo*. They also allow the preclinical testing of novel therapeutic approaches.

In the presented series of articles, we aimed at covering the most recent development in the field of HIS mouse models with a particular focus on the application of immunotherapies. The review by Martinov et al. provides up-to-date information on current mouse models that have a humanized hemato-lymphoid system. The authors discuss the underlying principles of these models and point out outstanding challenges to build the next generation of mice with a human hematopoietic and immune system. Importantly, this review also provides guidance on how to identify humanized mouse models suited for specific research investigations. In another review, Mian et al. focus on humanized mouse models to investigate cancer immunotherapy by first giving an overview of recently developed models. In particular, they discuss challenges related to the development of specific human immune cell types and link the assessment of cancer immunotherapies with specific humanized mouse models for preclinical *in vivo* testing.

## Novel Approaches to Improve Human Hematopoiesis in Mice

Hematopoiesis occurs primarily in the bone marrow, where the hematopoietic stem and progenitor cell (HSPC) undergo expansion and multilineage differentiation. This process is regulated by a specific microenvironment called the hematopoietic stem cell niche. To establish HIS mice, opening the niche by, e.g., sub-lethal irradiation, is required for the stable engraftment of human HSPCs in the physical space. Mice engineered to carry a mutation in the *Kit* gene allow the engraftment of human HSPCs without irradiation due to the reduced competition of human HSPCs with mouse HSPCs for niche space. Hess et al. investigated the engraftment potential of various hematopoietic stem cell sources in non-irradiated NOD.B6 *scid Il2rg* ^-/-^ *Kit* ^W41/W41^ (NBSGW) mice, which carry a hypomorphic mutation of the *KIT* gene. They show that the human engraftment level in mice transplanted with mobilized adult peripheral blood stem cells is significantly higher than in mice transplanted with human cord blood or bone marrow. The different HSPC sources also yielded different hematopoietic lineage engraftment patterns. However, the transplanted human cells lost the long-term engraftment potential *in vivo*. Adigbli et al. also used non-irradiated NBSGW mice, demonstrating engraftment of multiple human lymphoid and myeloid cell lineages after transplantation with CD133+ HSPCs. Moreover, NBSGW mice supported the development of human red blood cells when endogenous mouse macrophages were removed. Combined, the two studies provide useful information on the utility of the NBSGW mouse model for studying human hematopoiesis *in vivo*.

## Novel Tumor-Bearing Mouse Models to Evaluate the Interaction Between Human Tumors and Immune Cells

Immune checkpoint inhibitors (ICI) are widely used in the treatment of solid tumors. However, a preclinical model to evaluate the anti-effect of ICI has not been established yet. In the methods paper published by Marín-Jiménez et al., the authors provide a very detailed description of their extensive experience with testing immunotherapies in human cord blood-derived Balb/c Rag2^-/-^ *Il2rg* ^-/-^ *Sirpa* ^NOD^ mice. They investigated the response to ICI in various human solid tumors. Their work demonstrates that patient-derived xenografts are suited to investigate immunotherapies based on T-cell responses and constitutes an important resource for the development of preclinical models. Along these lines, Wunderlich et al. developed a preclinical model for pediatric and adolescents and young adults refractory B lymphoblastic leukemia (B-ALL) using NOD *scid Il2rg* ^-/-^ (NSG)-hSCF/IL3/GM-CSF^Tg^ or NOD Rag1^-/-^ *Il2rg* ^-/-^ (NRG)-hSCF/IL3/GM-CSF^Tg^ mice. Mice were “pre-engrafted” with human cord blood in order to generate functional effector T-cells. They confirmed the clinical efficacy of blinatumomab and also showed that interestingly the application of pembrolizumab reduced the B-ALL burden. Importantly, mice which were not pre-engrafted with human T-cells were unresponsive to treatment. Finally, they observed a synergistic effect of the combined treatment with blinatumomab and pembrolizumab. The combination led to an increased number of mice which lacked minimal residual disease and to prolonged survival supporting further investigation of this dual immunotherapeutic approach. Similarly, Qiao et al. developed a human tumor cell-line derived xenograft model for non-small cell lung cancer in order to test ICI. For this purpose, human PBMCs were transplanted before the tumor injection. They could show that the lactate dehydrogenase inhibitor oxamate and pembrolizumab act synergistically against tumor cells by increasing the number of CD8+ T-cells that infiltrate the tumor microenvironment.

Volk et al. used a model to mimic Epstein Baar virus (EBV)-associated post-transplant lymphoproliferative disorder (EBV-PTLD) by the infection of fully humanized NRG mice with EBV. They demonstrated that pembrolizumab induced expansion of EBV-positive tumor cells and infiltration of EBV-positive tumor cells into the central nervous system. This result is likely due to the emergence of dysfunctional T cells and may reflect clinical observations with spreading of EBV after ICI treatment.

## Immune Cell-Specific Anti-Tumor Responses Using HIS Mouse Models

The technical advances in the generation of humanized mice allow today to specifically investigate human immune cells *in vivo*. In the mini-review paper by Sun et al., the authors summarize recent advances with HIS mice that carry autologous tumor cells. They specifically focus on BLT humanized mice engrafted with human fetal liver CD34+ cells transduced with a MLL-AF9-GFP retrovirus to assess the response to immunotherapies in human leukemia, in particular anti-CD19 CAR T-cells.

In their original article, Katano et al. report the generation of human IL-15 transgenic NOD/Shi-*scid*/IL-2Rγ^null^ (NOG) mice, which lack the mouse Fcγ receptor and consequently do not produce antibody-dependent cellular cytotoxicity (ADCC). This allowed the authors to specifically examine the contribution of human NK cell-mediated ADCC to anti-tumor responses. Therefore, this new model may prove useful for the preclinical testing of antibody-based approaches known to induce ADCC.

Maser et al. studied the anti-tumor effect of plasmacytoid dendritic cells (pDCs) in NOG-hIL3/GM-CSF^Tg^ mice, which supported the development of human pDCs. In certain tumor models, pDCs were recruited to tumors growing in mice. These intratumoral pDCs were functional as they produced type I interferon (IFN) in response to Toll-like receptor stimulation, which may prove beneficial for promoting NK cell-mediated anti-tumor activity.

Based on the identification of human monocyte-restricted progenitors, Izumi et al. investigated the effect of an anti-CD64 dimeric antibody conjugated with pyrrolobenzodiazepine (dPBD) *in vivo* in NOG mice. They showed that the anti-CD64-dPBD antibody selectively depleted monocyte progenitors without affecting the mature compartment. This compound also prevented the development of chronic myelomonocytic leukemia in mice, a disease with high therapeutic need. Finally, they extended their work to solid tumors and showed that the anti-CD64-dPBD antibody led to tumor growth regression by eliminating the tumor-associated macrophage population.

The review by Serr et al. summarizes the possible use of HIS mice to investigate and develop immunotherapies based on regulatory T-cells (Tregs). Tregs are known to facilitate tumor growth. The authors propose to use Treg inhibition to enhance anti-tumor responses in personalized humanized mouse models that are reconstituted with the patient’s immune system and tumor cells.

Standard immunodeficient mice weakly support the development of human innate lymphoid cells (ILCs), in particular human NK-cells. Recent progress in the generation of humanized immunodeficient animals, in particular models that produce human IL-15, led to the development of human ILCs *in vivo*. In the review by Horowitz et al., the authors present humanized mouse models that can be used to study ILCs and potential therapeutic approaches that employ human ILCs against cancer. This review provides an excellent overview about the development of ILC-based cancer immunotherapy.

## Unique Applications for Specific Research Questions Using HIS Mice

Flahou et al. review the use of HIS mice in the context of induced pluripotent stem cells (iPSC)-derived therapies for tissue regeneration. Here the authors suggest using humanized mouse models that develop human NK cells to evaluate the immunogenicity and rejection of transplanted iPSCs and their progeny. This could help to develop HLA-engineered iPSC-derived cells for universal clinical use.

Tumor necrosis factor (TNF) is a pro-inflammatory cytokine that controls inflammation. TNF inhibitors have been widely used in the clinic to treat autoimmune or inflammatory diseases. Gogoleva et al. review several humanized mouse models, which express human TNF in order to study human autoimmune diseases and the effect of TNF inhibitors on hematopoiesis *in vivo*.

Human γ-herpesviruses such as Epstein Barr virus (EBV) and Kaposi sarcoma-associated herpesvirus (KSHV) can induce lymphoproliferative diseases in humans. The immune response against γ-herpesviruses has been largely recapitulated in humanized mouse models. The review article by Münz focuses on the evaluation of immune responses to control pathologies induced by human γ-herpesviruses in humanized mouse models and discusses strategies for developing vaccines.

Furthermore, Yong et al. tested two different immunotherapeutics, including human interleukin-2 (Proleukin) and the anti-CD3 monoclonal antibody OKT3, in a humanized NSG model. Administration of Proleukin to humanized mice resulted in an increase of several pro-inflammatory cytokines and chemokines. Likewise, the use of OKT3 treatment in their model led to T-cell depletion along with an increase in several pro-inflammatory cytokines. This indicates that the presented models can reproduce the effect of these immunotherapeutics observed in patients.

## Conclusion

This Research Topic shows the usefulness of HIS mouse models in translational research related to several important human diseases and we are looking forward to future developments in this exciting research area.

## Author Contributions

All authors listed have made a substantial, direct, and intellectual contribution to the work, and approved it for publication. All authors contributed equally to the work.

## Funding

YS is supported by a Grant-in-Aid for Challenging Exploratory Research (20K21547) from the Ministry of Education, Culture, Sports, Science and Technology of Japan. TW is supported by a faculty-funded career position at Karolinska Institutet (2-1060/2018). APAT is supported by the Professor Dr. Max Cloëtta Foundation and the Swiss Cancer Research grant KFS-4875-08-2019.

## Conflict of Interest

The authors declare that the research was conducted in the absence of any commercial or financial relationships that could be construed as a potential conflict of interest.

## Publisher’s Note

All claims expressed in this article are solely those of the authors and do not necessarily represent those of their affiliated organizations, or those of the publisher, the editors and the reviewers. Any product that may be evaluated in this article, or claim that may be made by its manufacturer, is not guaranteed or endorsed by the publisher.

